# Evaluation of *Steinernema khuongi* and *Heterorhabditis downesi* as Biological Control Agents Against Four Stored Product Beetle Pests

**DOI:** 10.3390/insects17060534

**Published:** 2026-05-22

**Authors:** Angeliki Maria N. Matzavaki, Maria C. Boukouvala, Anna Skourti, Demeter Lorentha S. Gidari, Dionysios Ntinokas, Alexandros Dritsoulas, Ioannis O. Giannakou, Nickolas G. Kavallieratos

**Affiliations:** Laboratory of Agricultural Zoology and Entomology, Department of Crop Science, Agricultural University of Athens, 75 Iera Odos Street, 11855 Athens, Attica, Greece; stud121058@aua.gr (A.M.N.M.); mbouk@aua.gr (M.C.B.); annaskourti@aua.gr (A.S.); dlgidari@aua.gr (D.L.S.G.); dntinokas@aua.gr (D.N.); alexdritsi@gmail.com (A.D.); igiannakou@aua.gr (I.O.G.)

**Keywords:** entomopathogenic nematodes, *Tenebrio molitor*, *Tribolium* spp., *Trogoderma granarium*, larvae, maize, wheat

## Abstract

Storage insects consist a continuous global threat to food safety. In this study, the efficacy of the entomopathogenic nematodes (EPNs) *Steinernema khuongi* and *Heterorhabditis downesi* was evaluated against larvae of *Tribolium castaneum*, *Tribolium confusum*, *Tenebrio molitor*, and *Trogoderma granarium* in wheat and maize at six concentrations and two exposure intervals. Both EPNs exhibited higher virulence when applied in wheat than in maize. Generally, larval mortalities were higher under *H. downesi* treatments vs. *S. khuongi*, both in commodities and exposures. Notably, the tested EPNs caused high mortalities to *T. granarium* larvae (range, 88.9–92.2%) and *T. castaneum* larvae (range, 81.1–94.4%), respectively, at 10,000 IJs/mL in wheat vs. mortality ranges 72.2–77.8% and 74.4–87.8% in maize, respectively. In contrast, *T. confusum* larvae were tolerant to both EPNs. *Tenebrio molitor* larvae were tolerant to *S. khuongi* (<34.0% mortality) whereas susceptible to *H. downesi* (>83.3% mortality) at 10,000 IJs/mL in wheat and maize. These findings highlight the potential of *S. khuongi* and *H. downesi* as beneficial organisms against several stored-product insect pests.

## Simple Summary

Insects attacking stored products cause considerable economic losses worldwide through degradation of their quality and quantity. The need for sustainable control methods of noxious insect species in storages leads to the examination of entomopathogenic nematodes (EPNs) as alternatives to chemical insecticides. The present study focuses on two less-studied nematode species, *Steinernema khuongi* and *Heterorhabditis downesi*, aiming to evaluate for first time their ability to kill larvae of the important beetle pests, *Tribolium castaneum*, *Tribolium confusum*, *Tenebrio molitor*, and *Trogoderma granarium* on different grains. The results indicated that both EPN species were effective against the majority of the tested insect species, providing information for the integration of these natural enemies into stored product protection.

## 1. Introduction

Entomopathogenic nematodes (EPNs) are effective biological agents against a wide range of insect pests [1,2,3]. *Heterorhabditis* (Heterorhabditidae) and *Steinernema* (Steinernematidae) are the main genera parasitizing more than 100 insect species [4,5]. The biology of EPNs is based on a symbiotic relationship with bacteria hosted in their digestive system, determining their parasitic success. The genera *Photorhabdus* and *Xenorhabdus* are associated with Heterorhabditidae and Steinernematidae, respectively [6]. The widespread use of EPNs as biocontrol agents is related to several important advantages. They are ecologically safe since they do not cause side effects in the ecosystem, remain harmless to the non-target organisms or the health of humans, and characterized by motility, persistence or ability to reproduce within the host, ensuring long-term control of the target pests [7,8,9]. EPNs penetrate the body of the host as third-stage infective juveniles (IJs), i.e., the only free-living stage of their life cycle [10,11]. Penetration occurs through natural openings, such as the oral cavity, anus, and respiratory stigmata, while in some cases it occurs directly through the intersegmental membrane of the cuticle [10,12]. Once inside the host’s body, IJs release their symbiotic bacteria into the hemolymph [13,14]. These bacteria are the main drivers of IJs’ pathogenesis, as they release various bioactive substances acting as immunosuppressive and insecticidal agents, including toxins, enzymes, and killer proteins [14,15]. All other life stages of EPNs develop inside the dead insect body, where they use the host’s tissues and symbiotic bacteria for nourishment [16,17,18]. Inside cadavers, EPNs can complete successive reproductive cycles (usually 1–3 generations) [19]. Finally, EPNs abandon the insect cadaver as IJs and actively seek new hosts to penetrate. In contrast, one of the main advantages of EPNs is their rapid action, as the pest can die within 24–48 h after penetration through septicemia and/or toxemia [16,20]. This feature gives EPNs a clear advantage over other biological agents, which usually require a longer period of time to complete the entomopathogenic action [11,21].

Insects attacking stored products cause extensive losses worldwide by degrading their commercial value through direct consumption, contamination with feces, and the creation of favorable conditions for the development of secondary fungal infections [22,23,24,25]. Among the most damaging species are the tenebrionids *Tenebrio molitor* L., *Tribolium confusum* Jacquelin du Val, and *Tribolium castaneum* (Herbst), as well as the quarantine dermestid *Trogoderma granarium* Everts [26,27,28,29]. All these species are globally distributed infesting a wide range of stored products (e.g., cereals, pulses, dried fruits, and products of animal origin) [30]. The potential of EPNs as agents for the control of insects in stored products is mainly attributed to their ability to actively locate hosts in cryptic habitats, as well as their compatibility with existing chemical insecticide application equipment [16,31]. The efficiency of EPNs in finding a host is based on different foraging strategies [32]. Ambushers have a waiting strategy, remaining essentially immobile until they approach a potential host. In contrast, cruisers are characterized by active movement in the environment to locate hosts, while EPNs that combine both strategies are known as intermediates [16,19,33,34]. Cruisers demonstrate greater efficiency in attacking immobile hosts on the ground, while ambushers are excellent at catching insects moving on the ground surface [19]. Regardless of the foraging strategy, host detection is achieved through sensory cues used by EPNs, such as hygrosensory, chemosensory, mechanosensory, or thermosensory [1,19,32].

*Heterorhabditis bacteriophora* Poinar (Rhabditida: Heterorhabditidae), *Steinernema feltiae* (Filipjev) and *Steinernema carpocapsae* Weiser (Rhabditida: Steinernematidae) are well investigated biological control agents of several stored-product insects. Previous studies confirm the high pathogenicity of these EPNs in larvae and/or adults of important coleopterans, such as *T. castaneum*, *T. molitor*, *Alphitobius diaperinus* (Panzer) (Tenebrionidae), *Sitophilus oryzae* (L.) (Curculionidae), *Oryzaephilus surinamensis* (L.) (Silvanidae), *Rhyzopertha dominica* (F.) (Bostrychidae), *Trogoderma variabile* Ballion (Dermestidae), and *T. granarium*, as well as Lepidoptera, including *Plodia interpunctella* (Hübner) and *Ephestia kuehniella* Zeller (Pyralidae) [16,34,35,36,37,38,39,40].

The recently described *Steinernema khuongi* Stock, Campos-Herrera, El-Borai, and Duncan (Panagrolaimomorpha: Steinernematidae) is native to the soil and environmental conditions that characterize Florida’s Flatwood citrus groves [41,42]. Genomic comparison allowed the identification of the symbiotic bacteria of *S. khuongi*, classifying them to species *Xenorhabdus poinarii* [42]. The total body length of IJs of *S. khuongi* is 1066 μm [41]. *Heterorhabditis downesi* Stock, Griffin, and Burnell (Rhabditida: Heterorhabditidae) is recognized as an important biological control agent [43]. The presence of *H. downesi* has been documented in the United Kingdom, Hungary, Denmark, Ireland, Italy, and Germany [44,45,46,47]. Morphometrically, the IJs of *H. downesi* are characterized by an average body length of 637 µm [45], while their body diameter is determined to be 18 µm [48]. The extensive search of the international literature revealed that there is no data on the virulence of *S. khuongi* and *H. downesi* against stored-product insects. Therefore, the present study examined their efficacy against *T. castaneum*, *T. confusum*, *T. molitor*, and *T. granarium* larvae under different EPN concentrations, exposure intervals in stored maize and wheat.

## 2. Materials and Methods

### 2.1. EPNs Cultures

Both EPNs were originally obtained from the Laboratory of Nematology, Citrus Research and Education Center, University of Florida (UF), USA, in 2022. Since then, the EPNs have been reared on fifth instar larvae of *Galleria mellonella* (L.) (Lepidoptera: Pyralidae) at the Laboratory of Agricultural Zoology and Entomology (LAGZE), Agricultural University of Athens (AUA). Both laboratories have confirmed the EPN populations with molecular tools. The UF laboratory used species-specific qPCR [49,50,51] and the AUA laboratory utilized the Sanger sequencing method using the universal primers AD58F (5′-TCGATGAAAAACGCGGCAA-3′) [52] and AB28R (5′-TATGCTTAAGTTCAGCGGGT-3′) [53], as forward and reverse primers, respectively.

The cultures of EPNs were maintained under laboratory conditions (25 °C, 50% relative humidity (RH)). Inoculation of the *G. mellonella* hosts was carried out in Petri dishes (12 cm diameter). Filter papers were placed in the inoculation dishes to cover the internal top and bottom of each dish completely. Then, 1000 infective juveniles (IJs) of *S. khuongi* or *H. downesi* were added as distilled water suspension to each dish separately to completely wet the filter papers. Finally, ten fifth-instar *G. mellonella* larvae were placed per dish and housed for 2–3 days in a controlled chamber under complete darkness at 25.0 °C and 80% RH. Dead larvae were transferred to White traps for the collection of newly hatched IJs. Specifically, a 10 cm dish lid with a filter paper was put upside down in a 20 cm dish, and the dead larvae were placed on the paper with tweezers. The 20 cm dish was filled with distilled water until the small lid barely floated in it. Before the tests, IJs’ viability was assessed through microscopic observation, and the populations of each EPN species that showed viability between 95–100% were selected [54]. IJs used in the bioassays were less than 5 d old.

### 2.2. Insect Cultures

The insect species examined were obtained from colonies kept in LAGZE under constant darkness and conditions of 65% RH and 30 °C. Whole wheat kernels and a mixture of wheat flour with 5% of brewer’s yeast were used as rearing media for *Tribolium* spp. and *T. granarium*, respectively, while *T. molitor* was grown on oat bran supplemented with fresh potato slices to promote moisture content, which were replaced at regular intervals. For the tests, larvae of *T. granarium* 4–6 mm, *T. molitor* 10–14 mm, and *Tribolium* spp. 1st-3rd instar were selected, after selection based on size using a combination of sieving and visual inspection under a stereomicroscope using ocular micrometer.

### 2.3. Commodities

Two grains were utilized in the experiments: a mixture of varieties of wheat (*Triticum durum* Desf.) and a mixture of varieties of maize (*Zea mays* L.), both uncontaminated and free of pesticides. Before the start of the bioassays, the moisture content of the wheat and maize was adjusted at 12.9% (by heating or adding distilled water) [55], as measured with a moisture meter (C-Pro grain, AgroLog, Søndersø, Denmark).

### 2.4. Bioassays

**Figure 1:**
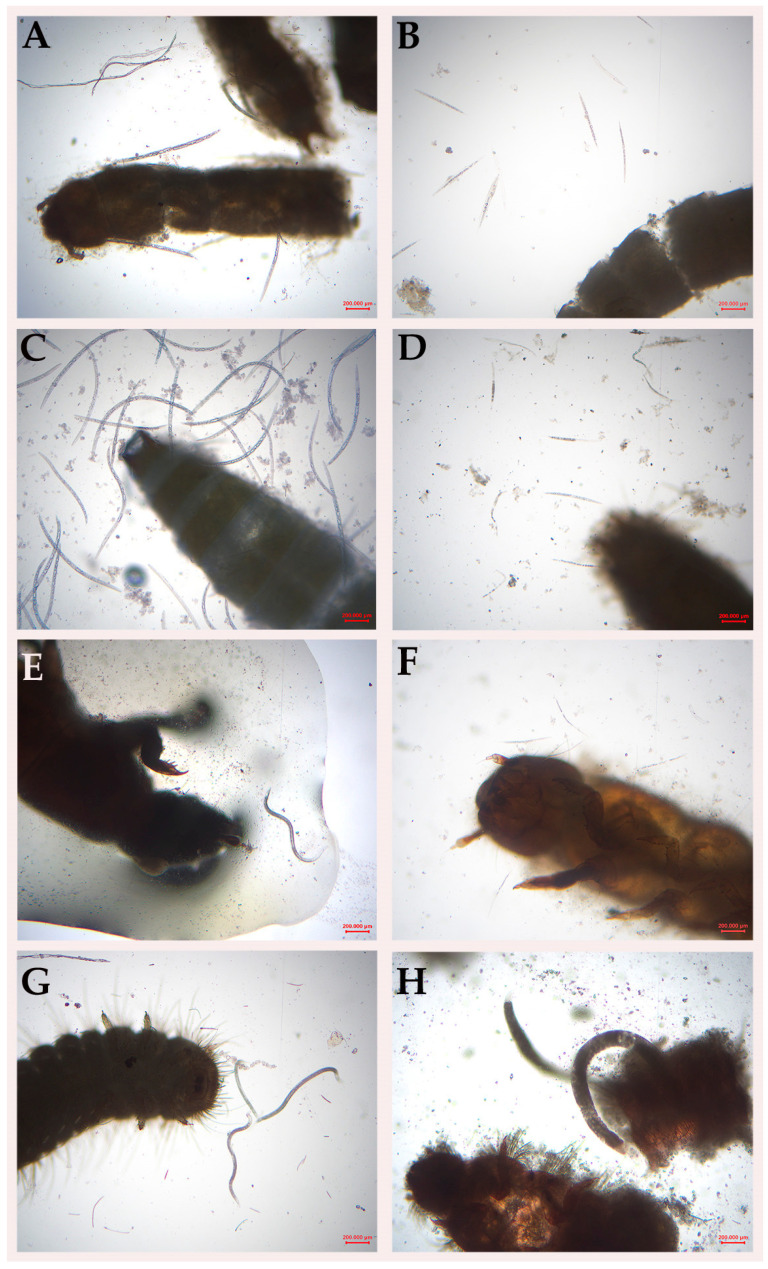
Images of dissected larvae of four insect species infected by *Steinernema khuongi* and *Heterorhabditis downesi*. (**A**,**B**) *Tribolium confusum*; (**C**,**D**) *Tribolium castaneum*; (**E**,**F**) *Tenebrio molitor*; (**G**,**H**) *Trogoderma granarium*. For each species, the left panels refer to the infection by *S. khuongi* (**A**,**C**,**E**,**G**) and the right panels by *H. downesi* (**B**,**D**,**F**,**H**). The images were acquired at the end of the experiments (8 days).

For the tests, Petri dishes with an 8 cm diameter and a 1.5 cm height were utilized. Ten grams of wheat or maize were put on each dish separately. Then, 1000 μL of nematode suspension (IJs) in distilled water of *H. downesi* or *S. khuongi* at concentrations of 50, 100, 500, 1000, 5000, and 10,000 IJs/mL were inoculated into each dish separately, using a micropipette, and finally shaken for approximately 1 min to achieve uniform dispersion of the nematode suspensions. Then, 10 larvae of each tested insect species, separately, were introduced into each dish. For the control dishes, only distilled water (1000 μL/dish) was used for inoculation. Larval mortality was recorded after 4 and 8 d post inoculation. Dead individuals were removed from the dishes after the 4 and 8 d mortality count and were washed meticulously to assure that all nematodes are coming from the hemolymph of the insect individuals. Dead larvae were collected from the dishes and subsequently dissected, as shown in Figure 1, to confirm the presence of nematodes [35]. All dead larvae gave nematodes after dissection. The experiments were conducted at 65% RH and 30 °C under continuous darkness. For each concentration (including control), nematode species, insect species, and commodity, three dishes (i.e., sub-replicates) were prepared. The whole experiment was totally replicated three times, as described above. In total, 1008 dishes were prepared for the experimentation (i.e., 3 dishes × 2 EPN species × 4 insect species × 2 commodities × 7 concentrations (6 EPN concentrations and control) × 3 replications). All the treatments within each replication were performed in the same day.

### 2.5. Statistical Analysis

No adjustment was required for control mortality, as it did not exceed 5%. Mortality data for each larval species treated with nematodes were logarithmically, log(x + 1), transformed to normalize variances [56,57]. Per insect species, the transformed data were subjected to repeated-measures analyses, with EPN species, EPN concentration, and commodity as the main effects. Exposure interval was the repeated factor, while mortality was the response variable [58]. The separation of means was performed using the test of Tukey HSD for multiple comparisons and the two-tailed *t*-test for paired comparisons, at a significance level of *p ≤* 0.05 [59]. Data analysis was conducted using JMP software version 16.2 [60].

## 3. Results

### 3.1. Mortality of Tenebrio molitor Larvae

All main effects were significant between exposure intervals. In addition, the main effects, exposure, and exposure × EPN were significant within exposure intervals (Table 1).

In general, mortality of *T. molitor* larvae was significantly higher in *H. downesi* treatments than in *S. khuongi*, in both commodities and exposure intervals, regardless of the concentration, except for 1000 IJs/mL in maize at the 4-day interval, where mortality was higher, but not significant (Table 2). Four days after nematode inoculation, mortality was very low at all *S. khuongi* concentrations, not exceeding 18.9 and 15.6% at 10,000 IJs/mL in treated wheat and maize, respectively. Concerning *H. downesi*, mortality rates were low to moderate, ranging from 16.7 to 55.6% in wheat and from 16.7 to 43.3% in maize.

Eight days after inoculation, mortality increased slightly at all *S. khuongi* concentrations, yet remaining low, ranging from 11.1 to 33.3% in wheat and from 10.0 to 30.0% in maize. Mortality caused by *H. downesi* ranged from low to moderate between 50 and 1000 IJs/mL in wheat (i.e., 24.4–63.3%) and between 50 and 5000 IJs/mL in maize (i.e., 24.4–60.0%). At concentrations ≥ 5000 IJs/mL in wheat and at 10,000 IJs/mL in maize, *H. downesi* killed > 81% of the treated larvae (Table 2).

### 3.2. Mortality of Tribolium castaneum Larvae

Between exposure intervals, all main effects were significant. Within exposure intervals, the main effects, exposure, and exposure x concentration were significant (Table 1). Generally, *H. downesi* caused higher larval mortality of *T. castaneum* than *S. khuongi* throughout the experiment. Additionally, in wheat mortality of *H. downesi* was significantly higher at 50, 100, 5000, and 10,000 IJs/mL than that of *S. khuongi*, in both exposure intervals (Table 3). In maize, significantly higher mortalities were recorded in *H. downesi* compared to *S. khuongi* at 500, 5000, and 10,000 IJs/mL after 4 d, and at 1000–10,000 IJs/mL after 8 d.

On day 4, mortality was low at 50–500 IJs/mL of *S. khuongi* in both commodities (range 24.4–44.4 in wheat and 23.3–41.1 in maize), whereas at concentrations ≥ 1000 IJs/mL, mortality exceeded 56% in both grains (Table 3). In the case of *H. downesi*, mortality was 92.2 and 80.0% at 10,000 IJs/mL in wheat and maize, respectively, while at concentrations ≤ 5000 IJs/mL, this EPN caused between 40% and 76.7% mortality of the exposed larvae in wheat. In maize, mortality was low at 50 IJs/mL (28.9%), while mortality ranged between 43.3 and 73.3% at the remaining concentrations.

Eight days post-inoculation, mortality remained low for *S. khuongi* at 50–100 IJs/mL in wheat (32.2–38.9%) and at 50–500 IJs/mL in maize (28.9–47.8%) (Table 3). However, at 10,000 IJs/mL mortality reached 81.1% in wheat and 74.4% in maize. Regarding *H. downesi*, mortality did not exceed 36.7% at 50 IJs/mL in maize, while in the rest of the tested concentrations, mortality was noted between 50.0 and 87.8%. In wheat, moderate mortality was recorded at concentrations ≤ 100 IJs/mL (46.7–55.6%), while at concentrations ≥ 500 IJs/mL, it surpassed 60.0%, reaching 94.4% at 1000 IJs/mL.

### 3.3. Mortality of Tribolium confusum Larvae

Between and within exposure intervals, all main effects were significant. Within exposure intervals, the interactions exposure × EPN and exposure × concentration were also significant (Table 1). In general, mortality of *T. confusum* larvae was significantly higher in *H. downesi* treatments than in *S. khuongi* during the entire experimentation, except for 50 IJs/mL in wheat and 5000 IJs/mL in maize, at both exposures (Table 4). Four days after inoculation, the performance of *S. khuongi* was low at all tested concentrations, not exceeding 42.2% in wheat and 38.9% in maize. Regarding *H. downesi*, larval mortality remained low at concentrations of 50–1000 IJs/mL in both grains, reaching 73.3% and 60.0% at 10,000 IJs/mL, in wheat and maize, respectively.

On the 8th day, mortality increased further in both EPN species and commodities. However, the mortality of *S. khuongi* remained low, especially at 50–500 IJs/mL, where the percentage of dead larvae reached 28.9% and 27.8%, in wheat and maize, respectively (Table 4). At 10,000 IJs/mL, this EPN killed 48.9% of larvae in wheat and 46.6% of larvae in maize. For *H. downesi*, at concentration ≤ 500 IJs/mL, mortality ranged from 32.2 to 46.7% in wheat and from 27.8 to 41.1% in maize, while at 10,000 IJs/mL, it killed 77.8% and 64.4% of the exposed larvae in wheat and maize, respectively.

### 3.4. Mortality of Trogoderma granarium Larvae

All main effects between exposures, as well as the main effects exposure and exposure x concentration within exposure intervals, were significant (Table 1). In both commodities, mortality caused by *H. downesi* was higher than that of *S. khuongi*. However, significant differences were recorded at 50, 100, 1000, and 5000 IJs/mL at 4 days and at 50, 100, and 5000 IJs/mL at 8 days in wheat. In maize, significant differences were found at 50 and 1000 IJs/mL after 4 days or 1000 IJs/mL after 8 days (Table 5). At 4 days post-inoculation, mortality caused by *S. khuongi* was low to moderate between 50 and 5000 IJs/mL in wheat, reaching 66.7%, while at 10,000 IJs/mL, mortality was 82.2%. A similar pattern was observed in maize for *S. khuongi*, with mortality ranging from 24.4% and 68.9%. Regarding *H. downesi*, larvae of *T. granarium* suffered 80.0% and 87.8% mortalities at 5000 and 10,000 IJs/mL, respectively, in wheat, while at concentrations ≤ 1000 IJs/mL, mortality did not surpass 68.9%. In maize, the percentage of dead larvae ranged between 34.4% and 72.2%.

On day 8, an increase in mortality was observed, following a similar trend observed in mortality rates on day 4. Specifically, mortality caused by *S. khuongi* remained low to moderate at concentrations ≤ 1000 and ≤5000 IJs/mL in wheat and maize, respectively, eventually reaching 88.9% in wheat and 72.2% in maize at 10,000 IJs/mL (Table 5). At 5000 and 10,000 IJs/mL, mortality caused by *H. downesi* was 86.7% and 92.2% in wheat and 73.3% and 77.8% in maize, respectively. At concentrations ≤ 1000 IJs/mL, the mortality rates ranged from 50.0% to 76.7% in wheat, while in maize they ranged from 40.0% to 66.7%.

## 4. Discussion

The findings of the present study revealed that *H. downesi* caused higher larval mortalities than *S. khuongi* across the tested scenarios (insect species and nematode concentration) to a great extent. Although the general mode of action of EPNs is similar, differences in the virulence of symbiotic bacteria between *Heterorhabditis* spp. and *Steinernema* spp. [6] may account for the higher efficacy observed for *H. downesi* compared to *S. khuongi*. Previous research has shown the superiority of *H. downesi* over other *Heterorhabditis* spp. or *Steinernema* spp. against the curculionid *Hylobius abietis* (L.) (Coleoptera) [61]. The authors recorded the highest reduction (i.e., 68–87%) in the adult emergence of *H. abietis* on stumps treated with *H. downesi* during the 3-year trials [61]. It is well known that the host species considerably influences the virulence of EPN. For example, within genus *Heterorhabditis*, Erdoğuş [39] observed that *H. bacteriophora* killed significantly more adults of *T. castaneum* compared to *S. carpocapsae* and *S. feltiae*, at 1000 IJs/mL, after 120 h of exposure under different temperature conditions (i.e., 15 and 25 °C). In contrast, Karanastasi et al. [38] reported the opposite results, with *S. carpocapsae* and *S. feltiae* being more efficacious than *H. bacteriophora* against larvae (large and small) of *T. granarium*. The fact that *H. downesi* exhibits elevated virulence to different host species reinforces the concept of biological control in the storage environment where multiple noxious insect species coexist [62].

The lower efficacy of *S. khuongi* observed in this study is supported by Filgueiras and Willett [63], who found that its virulence is highly variable and strain-dependent. Specifically, while the WEB strain of *S. khuongi* had a reduced probability of mortality of *Delia antiqua* (Meigen) (Diptera: Anthomyiidae) adults when emerging from infected pupae, the ARCA strain exhibited a greater virulence, with increased probabilities of mortality of the infected individuals [63]. Similarly, Ramos-Rodríguez et al. [16] found a variation in mortality caused by five strains of *H. bacteriophora* against larvae of *E. kuehniella* and *T. molitor*, ranging from 22 to 96% and 30 to 90%, respectively. Moreover, the strain Tok 20 of *H. bacteriophora* was less effective than the Kg11 strain against *T. castaneum* adults [39]. Given that different strains of the two species were not examined in the present study, these results highlight the need for further research to determine whether the higher performance of *H. downesi* over *S. khuongi* is a characteristic of the species or the specific strains.

Regardless of the grain type, concentration, and exposure, the tested beetle species exhibited carriable susceptibility to the EPNs. The results showed that *T. castaneum* larvae suffered the highest mortality rates by *H. downesi*, followed by *T. granarium*, *T. molitor*, and *T. confusum*. The susceptibility order of larval species to *S. khuongi* was *T. granarium* > *T. castaneum* > *T. confusum* > *T. molitor*. It is noteworthy that both EPNs caused high mortality (>88%) at 10,000 IJs/mL in *T. granarium* larvae. This finding is of particular importance, since this life stage of the insect is known for its exceptional tolerance to a wide range of contact insecticides [29,64,65]. High larval mortality of *T. granarium* has also been recorded previously. For instance, Karanastasi et al. [38] observed >91% mortality of *T. granarium* small larvae and >87% mortality of large larvae, 8 days after their exposure in wheat treated with *S. carpocapsae* and *S. feltiae* at 50,000 IJs/mL. The fact that similar or even higher levels of mortality were achieved in this study using *H. downesi* and *S. khuongi* highlights the ability of EPNs to effectively control a host that chemical insecticides fail to a great extent.

It should be noted that all insect species evaluated in the present study are external feeders [24]. This behavior likely enhances their exposure to entomopathogenic nematodes, facilitating host location and infection. In contrast, internal feeders may be less susceptible to EPNs due to their feeding habits which could limit nematode access, especially to those instars that are fully developed within kernels, e.g., larvae, pupae. Further research is required to evaluate the performance of these EPNs against various developmental stages of internal feeders.

Regarding genus *Tribolium*, a notable difference in susceptibility was observed between the two species. Larvae of *T. castaneum* were highly susceptible to both EPNs. In contrast, larvae of *T. confusum* tolerated both EPNs, especially *S. khuongi*. The high susceptibility of *T. castaneum* larvae to *Steinernema* spp., such as *Steinernemma riobrave* Cabanillas, Poinar and Raulston (Rhabditida: Steinernematidae), *S. carpocapsae*, and *S. feltiae*, has been documented by Ramos-Rodríguez et al. [16]. The authors recorded mortalities > 80% at concentrations ≥ 10 IJs/larva [16]. Similarly, elevated mortalities against *T. castaneum* larvae have also been reported for the thermotolerant Pakistani nematode strains of *Steinernema* spp. (i.e, 157, 211, 507, and Ham 10) and *Heterorhabditis* spp. (i.e., HAM-64 and 1743) [66]. Especially at the highest concentrations (i.e., 250 and 350 IJs/larva) of strains 157, Ham 10, HAM-64, and 1743, complete (100%) larval mortality was observed after 48 h of exposure [66]. Yağci [11] also found increased mortality against *T. castaneum* larvae when exposed to 1000 IJs/mL (the highest dose) of *S. feltiae* (90.9%) after 72 h and *H. bacteriophora* (76.8%) after 96 h at 25 °C. In contrast, in the case of *T. confusum*, the literature shows a wide variation in responses of larvae to EPNs, ranging from moderate mortalities (60.0% at 20,000 IJs/mL of *S. feltiae* at 20 and 30 °C, and 63.3% at 20,000 IJs/mL of *S. carpocapsae* at 20 °C) [35], to high mortalities (80 and 100% at 900 IJs/larva of *S. feltiae* UK 76 and Hawaii strains) [67]. Therefore, the present study supports the general trend of the bibliography and highlights the ability of both EPNs to cause high mortalities in *T. castaneum*, a fact that broadens the available biocontrol options for the management of this species of stored products.

*Tenebrio molitor* suffered elevated mortality rates (>83%) by *H. downesi* in both grains, further supporting the virulence of the genus *Heterorhabditis* in this host. For instance, *H. bacteriophora* suppressed larvae of *T. molitor* at >250 IJs/Petri dish, after 4 days at 25 °C [68]. Even at low concentrations (50 IJs/Petri dish), mortality was >83% [68]. Similarly, *Heterorhabditis beicherriana* Li, Liu, Nermuť, Půža and Mráček (Rhabditida: Heterorhabditidae) caused 70% mortality in *T. molitor* larvae after 40 h of exposure at 160 IJs/larva, while even lower doses (e.g., 80 IJs/larva) resulted in mortality of up to 50% in the same period of time [69].

Apart from using different EPN species/strains against stored-product insects and following different application methods related to abiotic or biotic conditions, the type of commodity that is treated with the EPNs seems to play a role in their insecticidal performance. In this study it is documented that wheat favored the effectiveness of both EPNs more than maize. This may be attributed to the size of the gaps between the kernels. Specifically, the spaces between the kernels of maize in a bulk are larger compared to that in wheat kernels [70,71]. EPNs exhibiting a “cruiser” search strategy, such as *H. downesi* and *S. khuongi* [72,73], are more effective when limited to small gaps between kernels. The low porosity of wheat facilitates host detection, increasing the probability of encountering and successfully infecting moving insects into the reduced spaces under the presence of EPNs.

## 5. Conclusions

The current research effort showed that under certain combinations of *H. downesi* or *S. khuingi* concentrations, exposures, commodity larvae of almost all tested species faced high mortality rates. This fact highlights that both EPNs are able to move and locate their targets within cereal grains under complex storage conditions. Therefore, *H. downesi* and *S. khuingi* form effective biological control agents, underscoring the need to be further evaluated against additional stored-product pests and their life stages on different grain commodities.

## Figures and Tables

**Table 1:** MANOVA parameters for main effects and associated interactions for mortality levels of *Tenebrio molitor, Tribolium castaneum, Tribolium confusum,* and *Trogoderma granarium* larvae between and within exposure intervals (for all species, error df = 192).

		*T. molitor*		*T. castaneum*		*T. confusum*		*T. granarium*	
Between exposure intervals									
Source	df	*F*	*p*	*F*	*p*	*F*	*p*	*F*	*p*
Intercept	1	4989.6	<0.01	57,323.1	<0.01	19,184.1	<0.01	60,207.1	<0.01
EPN	1	190.4	<0.01	39.9	<0.01	79.1	<0.01	34.4	<0.01
Commodity	1	4.6	0.03	8.4	0.01	15.2	0.01	25.1	<0.01
Concentration	5	24.2	<0.01	84.4	<0.01	37.2	<0.01	66.7	<0.01
EPN × commodity	1	0.3	0.60	0.5	0.46	0.7	0.40	0.4	0.56
EPN × concentration	5	1.2	0.32	1.3	0.26	1.5	0.20	1.4	0.22
Commodity × concentration	5	0.2	0.96	0.5	0.79	1.5	0.20	0.1	1.00
EPN × commodity × concentration	5	0.2	0.97	0.4	0.87	2.0	0.08	0.1	0.98
Within exposure intervals									
Source									
Exposure	1	284.5	<0.01	92.7	<0.01	68.7	<0.01	169.6	<0.01
Exposure × EPN	1	10.1	0.01	0.2	0.63	5.4	0.02	1.7	0.20
Exposure × commodity	1	1.6	0.21	0.4	0.55	1.7	0.19	0.3	0.60
Exposure × concentration	5	1.0	0.41	5.2	0.01	2.4	0.05	4.3	0.01
Exposure × EPN × commodity	1	0.3	0.61	0.6	0.43	2.0	0.16	0.1	0.88
Exposure × EPN × concentration	5	1.3	0.27	0.5	0.80	0.4	0.82	0.6	0.69
Exposure × commodity × concentration	5	0.2	0.98	0.1	0.99	0.6	0.72	0.9	0.50
Exposure × EPN × commodity × concentration	5	0.4	0.82	0.8	0.53	1.0	0.42	0.7	0.63

**Table 2:** Mean (%) mortality ± standard errors (SE) of *Tenebrio molitor* larvae after 4 and 8 days in wheat and maize treated with *Heterorhabditis downesi* and *Steinernema khuongi* at six concentrations. Within each column, per exposure interval, means followed by the same lowercase letters are not significantly different (df = 5, 53; Tukey HSD test at *p* = 0.05). Within each row, per commodity, asterisks indicate significant differences (df = 16; two-tailed *t*-test at *p* = 0.05).

Exposure	4 Days							
Commodity	Wheat				Maize			
Concentration	*H. downesi*	*S. khuongi*	*t*	*p*	*H. downesi*	*S. khuongi*	*t*	*p*
50 IJs/mL	16.7 ± 1.7 d *	4.4 ± 1.8 b	−4.1	0.01	16.7 ± 2.4 b *	3.3 ± 1.7 b	−4.7	0.01
100 IJs/mL	22.2 ± 2.2 cd *	6.7 ± 1.7 ab	−3.6	0.01	18.9 ± 2.0 b *	5.6 ± 1.8 ab	−3.7	0.01
500 IJs/mL	27.8 ± 3.2 bc *	10.0 ± 2.4 ab	−3.1	0.01	22.2 ± 2.2 b *	8.9 ± 2.0 ab	−3.0	0.01
1000 IJs/mL	37.8 ± 2.8 ab *	13.3 ± 2.4 ab	−3.7	0.01	24.4 ± 3.8 ab	10.0 ± 1.7 ab	−1.6	0.13
5000 IJs/mL	41.1 ± 3.9 ab *	14.4 ± 2.4 ab	−3.5	0.01	33.3 ± 4.1 ab *	13.3 ± 2.9 ab	−2.7	0.02
10,000 IJs/mL	55.6 ± 2.9 a *	18.9 ± 1.1 a	−11.7	<0.01	43.3 ± 3.3 a *	15.6 ± 1.8 a	−7.4	<0.01
*F*	18.9	4.0			3.9	3.9		
*p*	<0.01	0.01			0.01	0.01		
	8 Days							
50 IJs/mL	24.4 ± 2.4 e *	11.1 ± 2.6 b	−2.6	0.02	24.4 ± 4.4 d *	10.0 ± 2.9 b	−2.6	0.02
100 IJs/mL	45.6 ± 2.4 d *	13.3 ± 1.7 ab	−10.3	<0.01	35.6 ± 2.9 c *	12.2 ± 1.5 ab	−8.0	<0.01
500 IJs/mL	51.1 ± 5.6 cd *	22.2 ± 4.3 ab	−2.8	0.01	45.6 ± 2.4 bc *	20.0 ± 3.3 a	−5.4	<0.01
1000 IJs/mL	63.3 ± 1.7 bc *	26.7 ± 3.3 ab	−6.5	<0.01	47.8 ± 4.7 bc *	22.2 ± 2.8 a	−4.5	0.01
5000 IJs/mL	81.1 ± 3.9 ab *	28.9 ± 4.2 ab	−3.2	0.01	60.0 ± 5.8 ab *	27.8 ± 4.3 a	−3.9	0.01
10,000 IJs/mL	91.1 ± 3.5 a *	33.3 ± 2.9 a	−9.8	<0.01	83.3 ± 4.1 a *	30.0 ± 2.9 a	−9.5	<0.01
*F*	42.6	3.1			17.0	6.5		
*p*	<0.01	0.02			<0.01	0.01		

**Table 3:** Mean (%) mortality ± standard errors (SE) of *Tribolium castaneum* larvae after 4 and 8 days on wheat and maize treated with *Heterorhabditis downesi* and *Steinernema khuongi* at six concentrations. Within each column, per exposure interval, means followed by the same lowercase letters are not significantly different (df = 5, 53; Tukey HSD test at *p* = 0.05). Within each row, per commodity, asterisks indicate significant differences (df = 16; two-tailed *t*-test at *p* = 0.05).

Exposure	4 Days							
Commodity	Wheat				Maize			
Concentration	*H. downesi*	*S. khuongi*	*t*	*p*	*H. downesi*	*S. khuongi*	*t*	*p*
50 IJs/mL	40.0 ± 3.3 e *	24.4 ± 3.8 d	−2.9	0.01	28.9 ± 4.2 d	23.3 ± 3.3 d	−0.9	0.37
100 IJs/mL	48.9 ± 4.6 de *	35.6 ± 2.9 c	−2.3	0.04	43.3 ± 4.7 c	33.3 ± 4.1 cd	−1.5	0.15
500 IJs/mL	55.6 ± 4.1 cd	44.4 ± 3.8 bc	−2.0	0.07	52.2 ± 2.8 bc *	41.1 ± 3.5 bc	−2.5	0.02
1000 IJs/mL	67.8 ± 2.8 bc	63.3 ± 4.1 ab	−1.0	0.34	63.3 ± 3.3 ab	56.7 ± 2.9 ab	−1.5	0.15
5000 IJs/mL	76.7 ± 2.9 ab *	66.7 ± 3.3 a	−2.2	0.04	73.3 ± 2.4 ab *	62.2 ± 2.2 a	−3.4	0.01
10,000 IJs/mL	92.2 ± 1.5 a *	75.6 ± 2.9 a	−4.8	0.01	80.0 ± 3.3 a *	71.1 ± 2.0 a	−2.2	0.05
*F*	21.1	24.4			20.3	23.9		
*p*	<0.01	<0.01			<0.01	<0.01		
	8 Days							
50 IJs/mL	46.7 ± 4.4 c *	32.2 ± 4.3 d	−2.3	0.04	36.7 ± 5.5 d	28.9 ± 4.2 d	−1.1	0.30
100 IJs/mL	55.6 ± 5.3 bc *	38.9 ± 3.5 cd	−2.3	0.04	50.0 ± 5.3 cd	37.8 ± 4.9 cd	−1.5	0.15
500 IJs/mL	60.0 ± 3.7 bc	50.0 ± 5.0 bc	−1.7	0.11	56.7 ± 1.7 bc	47.8 ± 4.0 bc	−2.0	0.06
1000 IJs/mL	73.3 ± 3.3 ab	67.8 ± 4.0 ab	−1.1	0.29	70.0 ± 2.4 ab *	62.2 ± 2.2 ab	−2.4	0.03
5000 IJs/mL	82.2 ± 3.2 a *	70.0 ± 4.4 a	−2.4	0.03	75.6 ± 2.4 ab *	66.7 ± 3.3 ab	−2.2	0.04
10,000 IJs/mL	94.4 ± 1.8 a *	81.1 ± 3.1 a	−3.7	0.01	87.8 ± 1.5 a *	74.4 ± 3.4 a	−3.6	0.01
*F*	13.8	20.2			16.9	15.9		
*p*	<0.01	<0.01			<0.01	<0.01		

**Table 4:** Mean (%) mortality ± standard errors (SE) of *Tribolium confusum* larvae after 4 and 8 days on wheat and maize treated with *Heterorhabditis downesi* and *Steinernema khuongi* at six concentrations. Within each column, per exposure interval, means followed by the same lowercase letters are not significantly different (df = 5, 53; Tukey HSD test at *p* = 0.05). Within each row, per commodity, asterisks indicate significant differences (df = 16; two-tailed *t*-test at *p* = 0.05).

Exposure	4 Days							
Commodity	Wheat				Maize			
Concentration	*H. downesi*	*S. khuongi*	*t*	*p*	*H. downesi*	*S. khuongi*	*t*	*p*
50 IJs/mL	26.7 ± 3.3 d	18.9 ± 2.6 d	−1.7	0.11	23.3 ± 2.4 c *	10.0 ± 2.9 b	−2.8	0.01
100 IJs/mL	31.1 ± 2.6 cd *	22.2 ± 2.2 cd	−2.6	0.02	28.9 ± 2.6 bc *	19.8 ± 2.6 a	−2.7	0.02
500 IJs/mL	44.4 ± 6.0 bc *	24.4 ± 1.8 bcd	−3.5	0.01	36.7 ± 3.3 abc *	22.2 ± 3.2 a	−3.0	0.01
1000 IJs/mL	47.8 ± 2.2 abc *	32.2 ± 2.8 abc	−4.4	0.01	41.1 ± 4.2 ab *	28.9 ± 2.6 a	−2.3	0.04
5000 IJs/mL	57.8 ± 6.0 ab *	36.7 ± 3.3 ab	−2.5	0.02	51.1 ± 7.0 ab	32.2 ± 2.8 a	−1.6	0.12
10,000 IJs/mL	73.3 ± 5.3 a *	42.2 ± 2.8 a	−5.5	<0.01	60.0 ± 6.2 a *	38.9 ± 3.5 a	−2.9	0.01
*F*	12.5	10.8			7.0	9.3		
*p*	<0.01	<0.01			<0.01	<0.01		
	8 Days							
50 IJs/mL	32.2 ± 3.2 d	23.3 ± 2.9 d	−2.0	0.07	27.8 ± 1.5 c *	13.3 ± 2.4 b	−2.9	0.01
100 IJs/mL	37.8 ± 3.6 cd *	25.6 ± 2.4 cd	−2.9	0.01	32.2 ± 2.2 bc *	24.4 ± 1.8 a	−2.7	0.02
500 IJs/mL	46.7 ± 5.8 bcd *	28.9 ± 2.6 bcd	−2.8	0.01	41.1 ± 3.5 abc *	27.8 ± 4.9 a	−2.2	0.04
1000 IJs/mL	52.2 ± 2.8 abc *	37.8 ± 4.3 abc	−2.8	0.01	46.7 ± 5.0 abc *	31.1 ± 2.6 a	−2.7	0.02
5000 IJs/mL	66.7 ± 6.5 ab *	43.3 ± 4.1 ab	−2.3	0.04	54.4 ± 7.7 ab	41.1 ± 1.1 a	−6.7	0.50
10,000 IJs/mL	77.8 ± 5.7 a *	48.9 ± 2.6 a	−4.8	0.01	64.4 ± 5.0 a *	45.6 ± 1.8 a	−4.1	0.01
*F*	9.9	9.2			6.5	10.0		<0.01
*p*	<0.01	<0.01			0.01	<0.01		

**Table 5:** Mean (%) mortality ± standard errors (SE) of *Trogoderma granarium* larvae after 4 and 8 days on wheat and maize treated with *Heterorhabditis downesi* and *Steinernema khuongi* at six concentrations. Within each column, per exposure interval, means followed by the same lowercase letters are not significantly different (df = 5, 53; Tukey HSD test at *p* = 0.05). Within each row, per commodity, asterisks indicate significant differences (df = 16; two-tailed *t*-test at *p* = 0.05).

Exposure	4 Days							
Commodity	Wheat				Maize			
Concentration	*H. downesi*	*S. khuongi*	*t*	*p*	*H. downesi*	*S. khuongi*	*t*	*p*
50 IJs/mL	38.9 ± 3.5 d *	27.8 ± 3.6 e	−2.3	0.04	34.4 ± 2.9 d *	24.4 ± 2.9 d	−2.4	0.03
100 IJs/mL	47.8 ± 3.2 cd *	36.7 ± 4.1 de	−2.2	0.04	40.0 ± 4.1 cd	34.4 ± 5.0 cd	−1.0	0.33
500 IJs/mL	63.3 ± 6.5 bc	47.8 ± 3.2 cd	−1.5	0.16	50.0 ± 2.4 bc	42.2 ± 3.6 bc	−1.9	0.08
1000 IJs/mL	68.9 ± 3.5 ab *	54.4 ± 2.9 bc	−3.1	0.01	58.9 ± 2.6 ab *	47.8 ± 4.0 abc	−2.4	0.03
5000 IJs/mL	80.0 ± 2.4 ab *	66.7 ± 3.7 ab	−2.9	0.01	63.3 ± 3.3 ab	58.9 ± 3.5 ab	−0.9	0.37
10,000 IJs/mL	87.8 ± 3.6 a	82.2 ± 2.8 a	−1.2	0.27	72.2 ± 3.6 a	68.9 ± 3.5 a	−0.7	0.52
*F*	14.8	26.9			17.7	13.9		
*p*	<0.01	<0.01			<0.01	<0.01		
	8 Days							
50 IJs/mL	50.0 ± 4.4 c *	34.4 ± 3.4 e	−3.0	0.01	40.0 ± 4.1 c	30.0 ± 3.3 c	−1.7	0.11
100 IJs/mL	57.8 ± 4.9 bc *	44.4 ± 2.9 d	−2.1	0.05	44.4 ± 5.6 bc	41.1 ± 4.2 bc	−0.4	0.73
500 IJs/mL	68.9 ± 7.7 abc	53.3 ± 3.7 cd	−1.2	0.26	56.7 ± 2.9 ab	48.9 ± 3.9 ab	−1.7	0.12
1000 IJs/mL	76.7 ± 5.0 ab	64.4 ± 2.9 bc	−2.0	0.08	66.7 ± 2.9 a *	54.4 ± 5.0 ab	−2.1	0.05
5000 IJs/mL	86.7 ± 3.7 a *	73.3 ± 3.3 ab	−2.8	0.02	73.3 ± 2.9 a	64.4 ± 4.4 a	−1.9	0.08
10,000 IJs/mL	92.2 ± 2.8 a	88.9 ± 2.6 a	−0.9	0.40	77.8 ± 4.0 a	72.2 ± 4.0 a	−1.0	0.35
*F*	7.7	31.7			13.5	11.8		
*p*	<0.01	<0.01			<0.01	<0.01		

## Data Availability

Data are available within the article.
